# Supplementary material for: Enzymatic Pretreatment with Laccases from Lentinus sajor-caju Induces Structural Modification in Lignin and Enhances the Digestibility of Tropical Forage Grass (Panicum maximum) Grown under Future Climate Conditions
Source: Int J Mol Sci. 2021 Aug 31;22(17):9445. doi: 10.3390/ijms22179445 (PMC8431176; doi:10.3390/ijms22179445)
Supplement: Supplementary file 1 [file ijms-22-09445-s001.zip › ijms-1356516-supplementary.pdf]

# Enzymatic pretreatment with laccases from *Lentinus sajor-caju* induces structural modification in lignin and enhances the digestibility of tropical forage grass (*Panicum maximum*) grown under future climate conditions

Emanuelle Neiverth de Freitas[1], Robson Carlos Alnoch[2], Alex Graça Contato[1], Karoline Maria V. Nogueira[1], Eduardo José Crevelin[3], Luiz Alberto B. de Moraes[3], Roberto Nascimento Silva[1], Carlos Alberto Martínez[2], and Maria de Lourdes T. M. Polizeli[1,2]

[1]Departamento de Bioquímica e Imunologia, Faculdade de Medicina de Ribeirão Preto, Universidade de São Paulo, Ribeirão Preto 14049-900, Brazil; emanuelleneiverthf@gmail.com (E.N.F.); alexgraca.contato@gmail.com (A.G.C.); karolmvnogueira@gmail.com (K.M.V.N); rsilvausp@gmail.com (R.N.S.)

[2] Departamento de Biologia, Faculdade de Filosofia, Ciências e Letras de Ribeirão Preto, Universidade de São Paulo, Ribeirão Preto 14050-901, Brazil; robsonalnoch@usp.br (R.C.A.); carlosamh@ffclrp.usp.br (C.A.M.); polizeli@ffclrp.usp.br (M.d.L.T.M.P)

[3] Departamento de Química, Faculdade de Filosofia, Ciências e Letras de Ribeirão Preto, Universidade de São Paulo, Ribeirão Preto 14050-901, Brazil; ejcrevelin@ffclrp.usp.br (E.J.C); luizmoraes@ffclrp.usp.br (L.A.B.M)

*Corresponding author: polizeli@ffclrp.usp.br* (M.d.L.T.M.P)

**Table S1. Enzymatic activities of crude extract of *Lentinus sajor- caju* CCB 020 cultivated in orange waste for 14 days.**

| **Enzymes** | **Activities (mU/mL)** |
|---|---|
| Laccase | 22,516 ± 627 |
| Manganese peroxidase | 178 ± 32 |
| Endo-1,4-xylanase | 2,970 ± 300 |
| Endoglucanase | 80 ± 20 |
| Mannanase | 2,050 ± 56 |
| endo-1,5-α-L-arabinanase | 180 ± 10 |
| β-glucanase | 130 ± 12 |
| β-D-xylosidase | 6.6 ± 0.3 |
| β-D-galactosidase | 9.8 ± 0.6 |
| β-D-glucosidase | 0.92 ± 0.05 |
| α-L-arabinofuranosidase | 1.33 ± 0.4 |
| Cellobiohydrolase | 8.5 ± 0.3 |

Substrates for enzymatic activity: 3-ethylbenzothiazoline-6-sulphonic acid- ABTS (Laccase), 2,6-dimethoxyphenol DMP (Manganese Peroxidase), xylan beechwood (Endo-1,4-xylanase), carboxymethylcellulose—CMC (Endoglucanase), locust bean (Mannanase), Debranched arabinan (Endo-1,5-α-L-arabinanase), β-glucan (β-glucanase), p-nitrophenyl-β-D-xylanopyranoside (β-D-xylosidase), p-nitrophenyl-β-D-galactopyranoside (β-D-galactosidase), p-nitrophenyl-β-D-glycopyranoside (β-D-glucosidase), p-nitrophenyl-α-L-arabinofuranoside (α-L-arabinofuranosidase), and p-nitrophenyl-β-D-cellobioside (Cellobiohydrolase).

**Table S2. The amino acid sequence of the putative laccase from *Lentinus sajor-caju*. The peptides corresponding to those identified by mass spectrometry are shown in bold. The mass profiles obtained were used in the TPP server platform (Uniprot database) with the XTandem peptide search engine and ProteinProphet.**

| >tr\|Q9HFT4\|Q9HFT4_PLESA Laccase 4 (Fragment) OS= *Lentinus (Pleurotus) sajor-caju* OX=50053 GN=Lac4 PE=2 SV=1 |
|---|
| *MFPGARILATLTLALHLLHGTHAAIGPAGN**MYIVNEDVSPDSFARSAVVAR**SVPATDPTP<br>ATASIPGVLVQGNKGDNFQLNVVNQLSDTTMLRTTSIHWHGFFQAGSSWADGPAFVTQCP<br>VASGDSFLYNFNVPDQAGTFWYHSHLSTQYCDGLRGPFVVYDPSDPHLSLYDIDNADTVI<br>TLEDWYHIVAPQNAAIPTPDSTLINGKGRYAGGPTSPLAIINVESNKRYRFRLVSMSCDP<br>NFTFSIDGHSLLVIEADAVNIVPITVDSIQIFAGQRYSFVLTANQAVDKYWIRANPNLGS<br>T**GFVGGINSAILRYAGATEDD**PTTTSSTSTPLLETNLVPLENPGAPGPPVPGGADININL<br>AMAFDFTTFELTINGVPFLPPTAPVLLQILSGASTAASLLPSGSIYELEANKVVEISMPA<br>LAVGGPHPFHLHGHTFDVIRSAGSTTYNFDTPARRDVVNTGTGAN |

**Table S3: Analysis of variance (ANOVA) for Central Composite Design (CCD) for sugar yields (g $L^{-1}$) of laccase pretreated biomass of *P. maximum* control group.**

| Source of variation | Degree of Freedom (DF) | Sum Square (SS) | Mean Square (MS) | $F_{test}$ | |
|---|---|---|---|---|---|
| | | | | $F_{calculated}$ | $F_{table}$ |
| **Regression** | 6 | 16.22 | 2.70 | **10.24** | **3.09**[a] |
| **Residue** | 11 | 2.90 | 0.26 | | |
| **Lack of fit** | 8 | 2.62 | 0.33 | **3.48** | **8.85**[b] |
| **Pure error** | 3 | 0.28 | 0.09 | | |
| **Total** | 17 | 19.12 | | | |

[a]*F* 95% = 6;11 ; [b]*F* 95% = 8;3

**Table S4: Analysis of variance (ANOVA) for Central Composite Design (CCD) for sugar yields (g $L^{-1}$) of laccase pretreated biomass of *P. maximum* eT+eC group.**

| Source of variation | Degree of Freedom (DF) | Sum Square (SS) | Mean Square (MS) | $F_{test}$ | |
|---|---|---|---|---|---|
| | | | | $F_{calculated}$ | $F_{table}$ |
| **Regression** | 7 | 24.89 | 3.56 | **20.60** | **3.14**[a] |
| **Residue** | 10 | 1.73 | 0.17 | | |
| **Lack of fit** | 7 | 1.61 | 0.23 | **6.17** | **8. 89**[b] |
| **Pure error** | 3 | 0.11 | 0.04 | | |
| **Total** | 17 | 26.1 | | | |

[a]*F* 95% = 7;10 ; [b]*F* 95% = 7;3

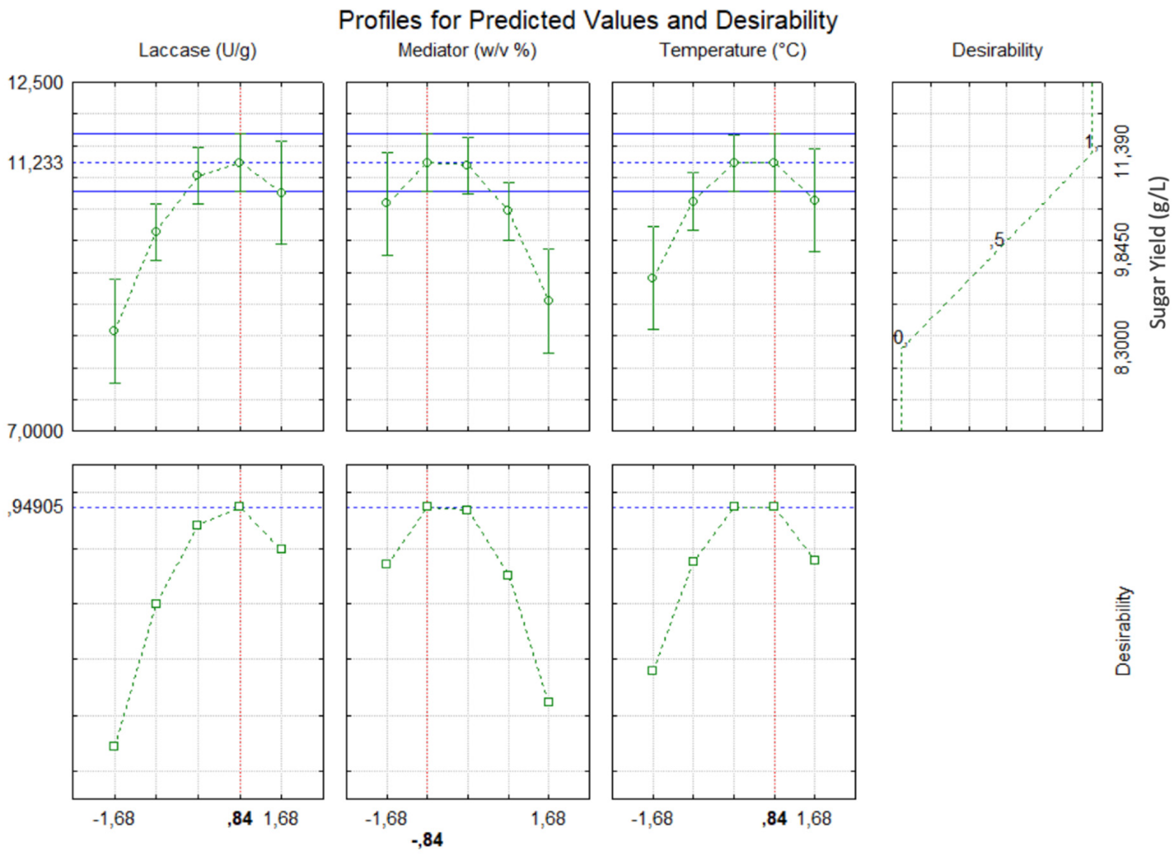


**Figure S1. Desirability profile of *P. maximum* control (C) group for Sugar Yield (g $L^{-1}$). Laccase, mediator, and temperature were the independent variables of CCD $2^3$.**

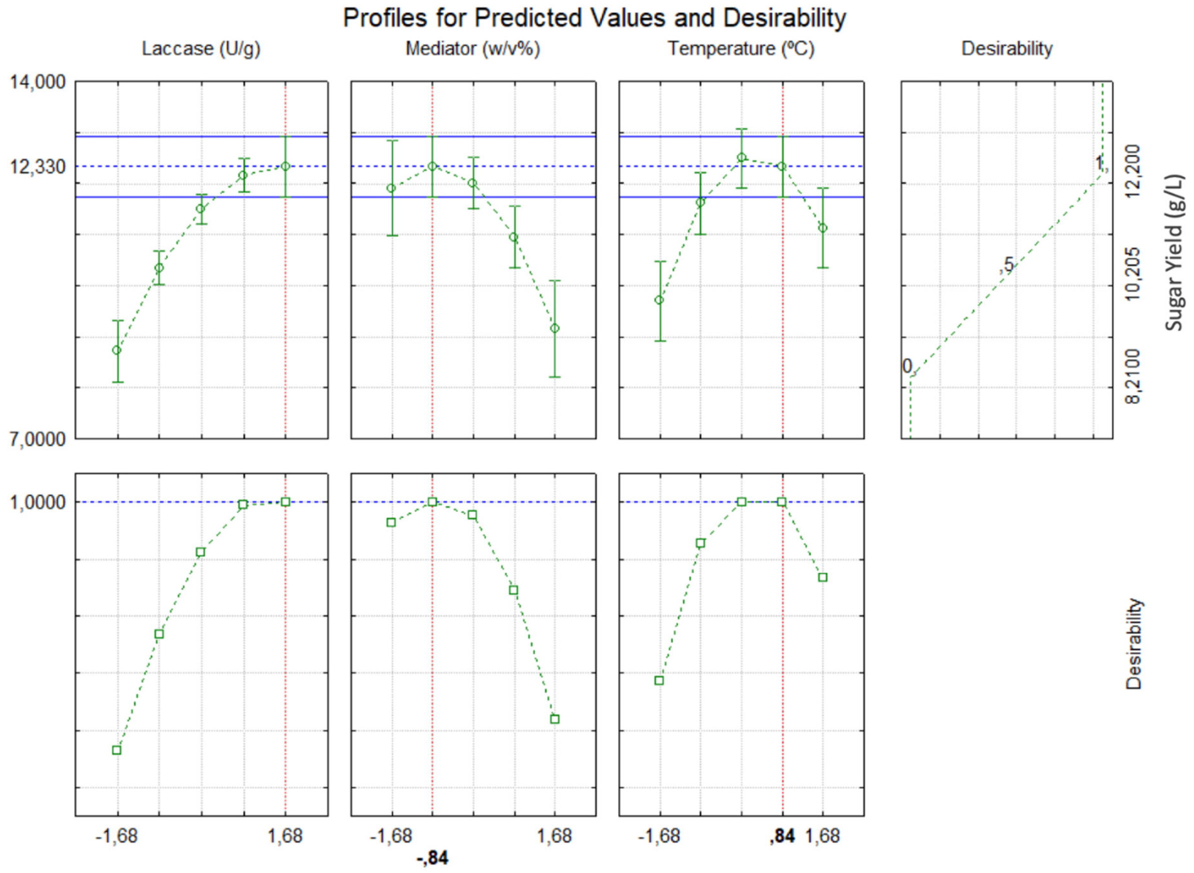


**Figure S2. Desirability profile of *P. maximum* elevated temperature+elevated $CO_2$ (eT+eC) group for Sugar Yield (g $L^{-1}$). Laccase, mediator, and temperature were the independent variables of CCD $2^3$.**

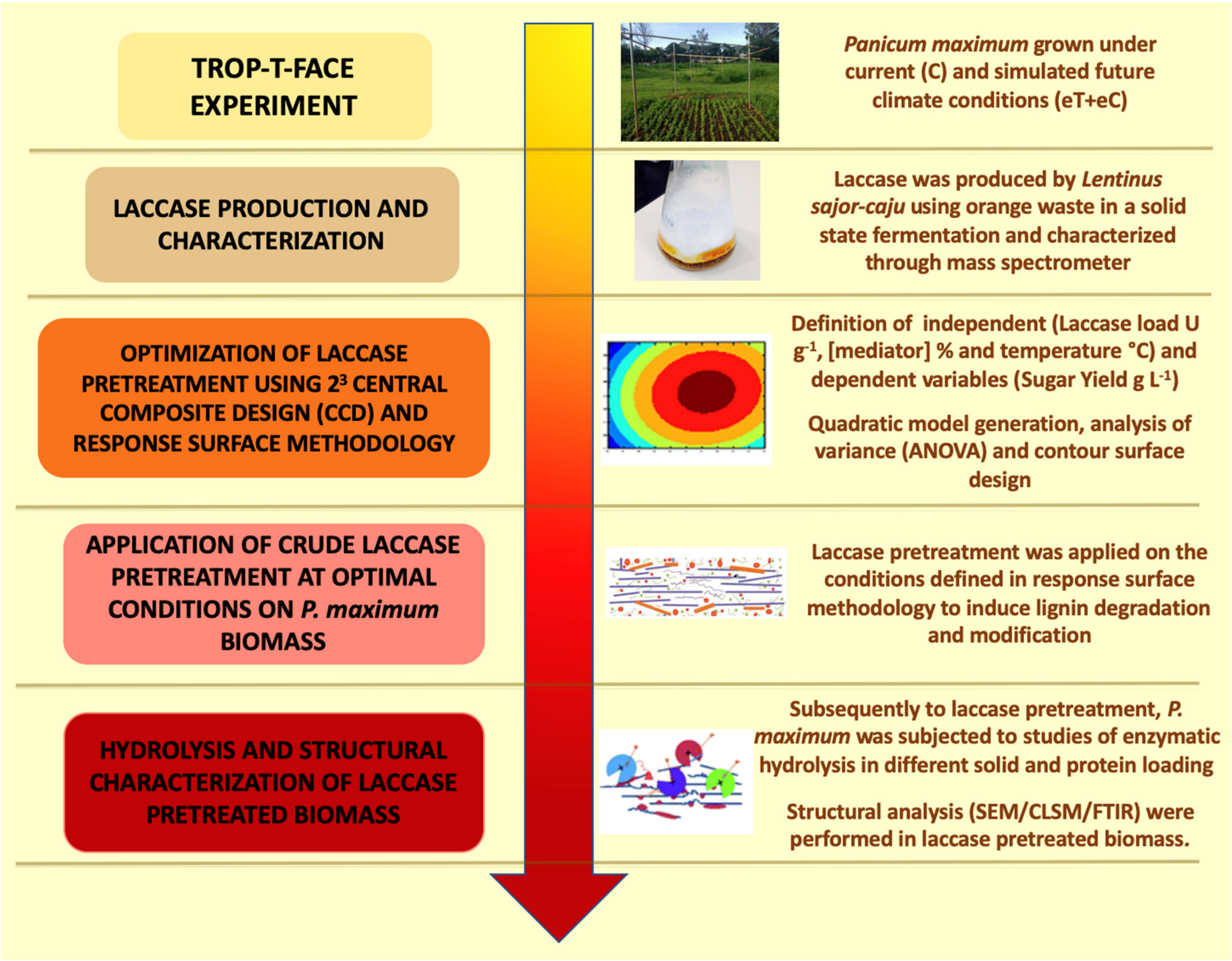


**Figure S3. Flow chart of the experimental design with the manly steps performed in the study.**
